# Single-cell ICP-MS for studying the association of inorganic nanoparticles with cell lines derived from aquaculture species

**DOI:** 10.1007/s00216-023-04723-6

**Published:** 2023-05-10

**Authors:** Cristian Suárez-Oubiña, Paloma Herbello-Hermelo, Natalia Mallo, María Vázquez, Santiago Cabaleiro, Ivone Pinheiro, Laura Rodríguez-Lorenzo, Begoña Espiña, Pilar Bermejo-Barrera, Antonio Moreda-Piñeiro

**Affiliations:** 1 Trace Element, Spectroscopy and Speciation Group (GETEE), Institute of Materials (iMATUS), Department of Analytical Chemistry, Nutrition and Bromatology, Faculty of Chemistry, Universidade de Santiago de Compostela, Avenida das Ciencias, s/n, 15782 Santiago de Compostela, Spain; 2 Centro Tecnológico del Cluster de la Acuicultura (CETGA), Punta Couso S-N, Ribeira, 15965 Spain; 3 INL-International Iberian Nanotechnology Laboratory, Av. Mestre José Veiga s/n, 4715-330, Braga, Portugal

**Keywords:** scICP-MS, Ammonia-based reaction cell, Titanium dioxide nanoparticles, Silver nanoparticles, Nanoparticle uptake in cells, Aquaculture species

## Abstract

The current research deals with the use of single-cell inductively coupled plasma-mass spectrometry (scICP-MS) for the assessment of titanium dioxide nanoparticle (TiO_2_ NP) and silver nanoparticle (Ag NP) associations in cell lines derived from aquaculture species (sea bass, sea bream, and clams). The optimization studies have considered the avoidance of high dissolved background, multi-cell peak coincidence, and possible spectral interferences. Optimum operating conditions were found when using a dwell time of 50 μs for silver and 100 μs for titanium. The assessment of associated TiO_2_ NPs by scICP-MS required the use of ammonia as a reaction gas (flow rate at 0.75 mL min^−1^) for interference-free titanium determinations (measurements at an m/z ratio of 131 from the ^48^Ti(NH)(NH_3_)_4_ adduct). The influence of other parameters such as the number of washing cycles and the cell concentration on accurate determinations by scICP-MS was also fully investigated. Cell exposure trials were performed using PVP-Ag NPs (15 and 100 nm, nominal diameter) and citrate-TiO_2_ NPs (5, 25, and 45 nm, nominal diameter) at nominal concentrations of 10 and 50 μg mL^−1^ for citrate-TiO_2_ NPs and 5.0 and 50 μg mL^−1^ for PVP-Ag NPs. Results have shown that citrate-TiO_2_ NPs interact with the outer cell membranes, being quite low in the number of citrate-TiO_2_ NPs that enters the cells (the high degree of aggregation is the main factor which leads to the aggregates being in the extracellular medium). In contrast, PVP-Ag NPs have been found to enter the cells.

**Graphical abstract:**

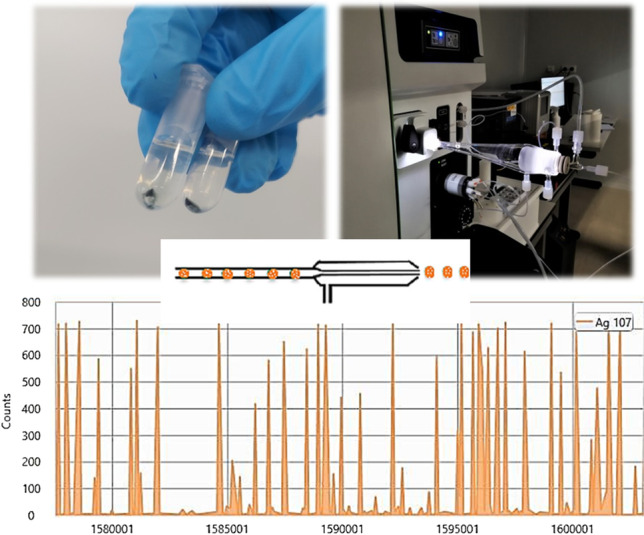

**Supplementary Information:**

The online version contains supplementary material available at 10.1007/s00216-023-04723-6.

## Introduction

The widespread use of nanoparticles (NPs) in several industrial sectors, including medicine, food, cosmetics, and even construction [1–4], has led to the presence of these new pollutants in the aquatic environment, and there is great concern about the risk for humans [5–7]. Among NPs, titanium dioxide nanoparticles (TiO_2_ NPs) and silver nanoparticles (Ag NPs) deserve special mention because of the huge use and applications: strong UV absorbance, photocatalytic capabilities, visible light transparency, iridescent qualities, and anti-bacterial activity for TiO_2_ NPs [8]; and catalytic activity, and optical and anti-bacterial properties for Ag NPs [9, 10]. The aquaculture sector could be affected by the increasing presence of NPs in the marine environment, and since this sector has experienced a continuous growth over the last decades [11, 12], concerns about potential hazards linked to emerging pollutants such as NPs are being considered to guarantee high-quality and safe products to the consumers.

As a preliminary step for elucidating pollutants bioaccumulation in cultured seafood, in vitro assays for assessing NPs association, either interaction with the cellular membrane or internalization, in cells are appealing approaches but, currently, there is not a consensual protocol for establishing this NPs-cells interaction [13–15]. Most analytical developments for elucidating metals internalization and NP interaction in/with cells require the analysis of large number of cells after several pre-treatments such as cellular lysis, extraction, and digestion procedures [16–19].

Single-cell inductively coupled plasma-mass spectrometry (scICP-MS) has opened a new area of research which allows the quantification of dissolved metals and inorganic NPs in single cells at ultra-low levels (attograms per cell). The mass of metal per cell, the mass distribution within a cell population, the concentration of metal- or nanoparticle-containing cells, and the number of NPs per cell can be determined using this cutting-edge technique. Among other high-quality features, scICP-MS can distinguish single cells and operates at a minimal sample uptake rate of 10 μL min^−1^, and analysis is performed close to native cell-state.

In scICP-MS, a suspension of cells must be nebulized using a nebulization system that allows cell integrity so that the single-particle ICP-MS (spICP-MS) principles can be used. As each cell enters the plasma, it is atomised, and the resulting ion-burst from the corresponding metal is detected by ICP-MS. Cells transfer into the plasma is one of the main challenges which faces scICP-MS. Cells transport system must keep the cell integrity and transfer the highest number of cells into the ICP-MS. Although several pneumatic nebulizers have been proposed for scICP-MS [16], microflow-based nebulizers in combination with special spray chambers guarantee cell integrity during the transport and hence, high transport efficiencies and low background signals. The special spray chambers have a dual make-up gas entrance specifically positioned to provide a tangential flow to the spray chamber walls, preventing cell collision and deposition onto the walls. Single-cell analysis by scICP-MS has been used for assessing the internalization of arsenite in A549 cells [17], copper in human red blood cells [18], copper-based algaecides in algae [20], and cisplatin in cancer cells [21]. Some scICP-MS applications have been also developed for elucidating NP association to cells, mainly gold nanoparticles (Au NPs) in microalgae, and in MCF-7, K562, and HeLa cells [22–24], Ag NPs in THP-1 monocytes and HepG2 cells [25, 26], biogenic selenium nanoparticles (Se NPs) in yeast [27], ZnO NPs in HepG2 cells [28], tellurium nanoparticles (Te NPs) in bacterial cells [29], and CdSeS quantum dots in Raw 264.7 cells [30].

In this sense, the aim of this work has been the optimization and application of scICP-MS to assess the association, either membrane adsorption or internalization, of PVP-Ag NPs and citrate-TiO_2_ NPs in cells from sea bass (*Dicentrarchus labrax*) kidney, sea bream (*Sparus aurata*) kidney, and clam (*Ruditapes philippinarum*) gills after several exposure trials. Optimization has implied the study of several parameters to avoid high dissolved background, multi-cell peak coincidence, and possible spectral interferences. The latter task is quite important for titanium determination, and the use of ammonia, previously used as a reaction gas in collision/reaction cell technologies for total titanium [31] and TiO_2_ NP [32] assessment by ICP-MS/MS and spICP-MS/MS, respectively, has been novelty used for scICP-MS analysis when assessing the titanium content at cells (monitoring the ^48^Ti(NH)(NH_3_)_4_ adduct at a m/z ratio of 131) [33, 34].

## Experimental

### Instrumentation

Analyses have been performed using a NexION 2000 quadrupole-based inductively coupled plasma mass spectrometer (PerkinElmer, Waltham, MA, USA), equipped with a quartz torch with a quartz injector (2.5 mm i.d.), triple cone equipment, and collision/reaction cell. The instrument is also equipped with the specialized Single Cell Micro DX autosampler and with high-efficiency introduction system consisting of a CytoNeb with PFA gas line nebulizer (PerkinElmer), fitted onto the Asperon spray chamber (PerkinElmer). Microjet adapter is positioned to the Asperon spray chamber for dual make-up gas inlet to create a tangential flow. All analyses were carried out using the Syngistix™ Single Cell Application Software Module for data collection and processing. Microwave-assisted acid digestions were performed using Ethos Easy Advanced Microwave Digestion System (Milestone, Sorisole, Italy). A Laborcentrifugen 2K15 (Sigma, Osterode, Germany) was used for centrifugation (washing of cell suspension) and a USC-TH ultrasound water bath (45 Hz, 80 W) from VWR International Eurolab S.L. (Barcelona, Spain) was used for dispersing NP standards before calibrations. Cell counting was performed in a counting Neubauer chamber–improved bright-line hemocytometer (Brand, Wertheim, Germany).

Electron microscopy (EM) characterization of exposed cells was performed using a JEOL JEM 1010 transmission electron microscope (TEM) operating at voltage of 100 kV for cells from clams and a scanning electron microscope (SEM) FEI Quanta 650 FEG, operating at high vacuum, an acceleration voltage of 5 kV, and spot size set at position 3 for kidney cells from sea bream. The cell samples for SEM analysis were coated with conductive carbon using an EM ACE600 coating system (Leica microsystems). The cell samples for TEM analysis were treated using a Leica EM TP Tissue processor and the ultrathin sections with a thickness of ~70 nm were prepared using a RMC Boecketer PowerTome PC ultramicrotome system.

### Reagents and standards

Ultrapure water (18.2 MΩ cm of resistivity) was obtained from a Milli-Q® IQ 7003 purification device system from Millipore (Bedford, MA, USA). Mono-elemental standards of ionic titanium [(NH_4_)_2_TiF_6_] and silver (AgNO_3_) were purchased from PerkinElmer. Hyperpure nitric acid 69% (w/v) and 33% (w/v) hydrogen peroxide were from Panreac (Barcelona, Spain). Phosphate-buffered saline (PBS) was from Thermo Fisher (Dublin, Ireland). NexION Setup Solution (Be, Ce, Fe, In, Li, Mg, Pb, U), 10 μg L^−1^, was from PerkinElmer. Glassware and plastic ware were decontaminated by soaking in 10% (v/v) nitric acid for at least 48 h. Material was then rinsed with ultra-pure water several times. Gold nanosphere dispersions were prepared from a N8151035 standard (nanoComposix, San Diego, CA, USA). The material consists of 49.6 ± 2.1 nm nanospheres (TEM diameter) and particle concentration of 9.89 × 10^6^ NPs mL^−1^ (2% RSD) obtained by spICP-MS. The nanospheres are covered by PEG carboxyl and they are suspended in aqueous 1 mM citrate. Silver NP dispersions used for calibration were prepared from bare (citrate) Ag NP standards (aqueous 2 mM sodium citrate) from nanoComposix. The standards were 60-nm Ag NPs (nominal diameter of 59 ± 6 nm obtained by TEM, mass concentration of 0.020 mg mL^−1^ obtained by ICP-MS, particle concentration of 1.8 × 10^10^ particles mL^−1^, and a hydrodynamic diameter of 64 nm); 40-nm Ag NPs (nominal diameter of 41 ± 5 nm obtained by TEM, mass concentration of 0.021 mg mL^−1^ obtained by ICP-MS, particle concentration of 5.4 × 10^10^ particles mL^−1^, and a hydrodynamic diameter of 44 nm); and 20-nm Ag NPs (nominal diameter of 20.8 ± 3.0 nm obtained by TEM, mass concentration of 0.021 mg mL^−1^ obtained by ICP-MS, particle concentration of 4.2 × 10^11^ particles mL^−1^, and a hydrodynamic diameter of 27 nm). Titanium dioxide NP stock dispersions (also used for calibration) were prepared from TiO_2_ suspensions (mixture of rutile and anatase, 99.5%) with particle size < 150 nm (volume distribution by dynamic light scattering) at 40 wt% in water, purchased from Sigma-Aldrich (Osterode, Germany). Regarding cell exposure trials, polyvinylpyrrolidone (PVP)-coated Ag NPs with primary nominal diameter of 15 nm and 100 nm and citrate-coated TiO_2_ NPs with a primary nominal diameter of 5 nm, 25 nm, and 45 nm were prepared from Ag NPs and TiO_2_ NPs from several suppliers. A complete description of the preparation of PVP-Ag NPs and citrate-TiO_2_ NP dispersions can be found in the “Preparation of PVP-Ag NPs and citrate-TiO_2_ NP dispersions used for cell exposure trials” section.

Ammonia (99.999%) reaction gas used in the reaction cell and 99.998% argon used for plasma generation, nebulization, and as auxiliary gas were supplied by Nippon Gases (Madrid, Spain).

Leibovitz medium (L15) and fetal bovine serum (FBS) suitable for cell growth, and ACS grade dimethyl sulfoxide (DMSO) were purchased from Thermo Fisher. Sodium cacodylate buffer was prepared by dissolving 98% sodium cacodylate trihydrate (Thermo Fisher) in distilled water and adjusting the pH to 7.2 with diluted hydrochloric acid prepared from technical grade 37% hydrochloric acid (Panreac). Karnovsky fixative was prepared at 2.0% (v/v) paraformaldehyde (96% extra pure) and 2.5% (v/v) glutaraldehyde (50% solution, ≥ 48.0 to ≤ 52.0%) from Thermo in 0.1 M sodium cacodylate buffer. Propylene oxide (ReagentPlus® ≥ 99%; Sigma-Aldrich, Merck Life Science, Algés, PT), osmium tetroxide solution (2% and 4% aqueous solution; Science Services, Munich, Germany), ethanol, and EMBed-812 epoxy resin kit (Science Services, Munich, Germany) were used for the fixation, staining, dehydration, and resin embedding of the cells for TEM analysis.

### Preparation of PVP-Ag NPs and citrate-TiO_2_ NP dispersions used for cell exposure trials

#### Polyvinylpyrrolidone (PVP)-coated Ag NPs with a diameter of 15 nm (15-nm Ag NPs)

Commercial PVP-coated Ag NP powder was purchased from SSNano (Houston, TX, USA; product code: 0127SH). The powder composition was 25% wt silver and 75% wt PVP. Ag NP stock dispersion at 6.2 g L^−1^ was prepared in ultrapure water by dispersing the powder for 15 min using a bath sonicator (37 kHz, 100%).

#### PVP-coated Ag NPs with a diameter of 100 nm (100-nm Ag NPs)

The 100-nm Ag NP stock dispersion with a concentration of 2.9 g L^−1^ was prepared from Ag ink containing 30% AgNPs with a diameter of 100 nm dispersed in ethylene glycol (Sigma-Aldrich). The ethylene glycol was removed by dialysis using a 12-kDa cellulose membrane against water. The purified Ag NPs was mixed with PVP (Mw = 40 kDa) solution to reach a Ag to PVP ratio of 1:3 wt:wt.

#### Citrate-coated TiO_2_ NPs with a primary size of 5 nm (5.0-nm TiO_2_ NPs)

Pristine 5-nm TiO_2_ NPs were purchased from Nanostructured & Amorphous Materials, Inc. (Katy, TX, USA; anatase, 5 nm size, 99%). The 5-nm TiO_2_ NP stock dispersion was prepared in ultrapure water by dispersing a mixture of trisodium citrate dehydrate and titanium dioxide powder at weight ratio of 1.5:1 wt:wt for 30 min using an ultrasonic probe (Branson Disintegrator Ultrasonic Model 450; 30-s pulse on/5-s pulse off, and 50% amplitude). The final concentration of citrate-coated TiO_2_ NPs was 13.3–15.5 g L^−1^ depending on the batch.

#### Citrate-coated TiO_2_ NPs with a primary size of 25 nm (25-nm TiO_2_ NPs)

Pristine 25-nm TiO_2_ NPs were supplied by Sigma-Aldrich (99.5% purity, mixture of rutile and anatase). The 25-nm TiO_2_ NP stock dispersion was prepared in ultrapure water by dispersing a mixture of trisodium citrate dehydrate and titanium dioxide powder at weight ratio of 0.8:1 wt:wt for 30 min using an ultrasonic probe (Branson Disintegrator Ultrasonic Model 450, 30-s pulse on/5-s pulse off, and 50% amplitude). The final concentration of citrate-coated TiO_2_ NPs was 13.3–15.5 g L^−1^ depending on the batch.

#### Citrate-coated TiO_2_ NPs with a primary size of 45 nm (45-nm TiO_2_ NPs)

Pristine 45-nm TiO_2_ NPs were purchased from Sigma-Aldrich (99.5% purity, mixture of rutile and anatase; nanoparticle size of < 100 nm (BET) and < 50 nm (XRD)) and were used without any further purification. The 45-nm TiO_2_ NPs were stabilized with trisodium citrate dehydrate aqueous solution reaching a weight ratio of 1:1.5 TiO_2_ to citrate. The mixture was dispersed for 30 min using an ultrasonic probe (Branson Disintegrator Ultrasonic Model 450; with 30-s pulse on/5-s pulse off, and 50% amplitude). The final concentration of citrate-coated TiO_2_ NPs was 13.3–15.5 g L^−1^ depending on the batch.

TEM analyses from several prepared Ag NPs and TiO_2_ NPs are shown in Figure S2 (electronic supplementary information, ESI).

#### Cytometry measurements

After appropriate dilution, 10 μL of cell suspensions was introduced in the chamber following manufacturer’s instructions and cells included in the 4 ruled areas of the corners were counted and the average number was accounted. Cells were stained with Trypan Blue classical method, to differentiate dead and alive cells. The final number of cells was calculated by multiplying average number by a factor of 10^4^ (manufacturer’s instructions) as well as by the dilution factor.

#### Cell culture conditions and pre-treatments for scICP-MS and EM analysis

Assays regarding exposure tests and cell toxicity to PVP-Ag NPs and citrate-TiO_2_ NPs for clam gills, and sea bass and sea bream kidney cells were carried out at the facilities of the Aquaculture Cluster Technology Centre (CETGA). The species (clam (*Ruditapes philippinarum*), sea bass (*Dicentrarchus labrax*), and sea bream (*Sparus aurata*)) followed the standard procedure for growth under controlled conditions until they were harvested. On the one hand, sea bass and sea bream specimens were fasted for 1 day before sampling, whereas the fasting period for clams was 3 days. The specimens were sacrificed after anaesthesia overdose, and the surface of the fish and the clam shells were disinfected with 70% ethanol. In the case of sea bass and sea bream, the kidney was removed under sterile conditions. The clams were opened by sectioning the anterior and posterior adductor muscles with a scalpel, and the entire contents of the clam’s belly were then removed under sterile conditions.

Both the kidney cells (sea bass and sea bream) and the entire contents of the soft tissues of clams were disaggregated, and the cells of interest were collected using a Percoll gradient, discarding impurities and non-viable cells. A Neubauer chamber was used to count total/viable cells. After counting, the cells were adjusted to the desired concentration, distributed in the wells, and loaded in multi-well plates for different exposures with NPs (size and concentrations). The cell culture was adjusted to a concentration of 4.0 × 10^6^ cell mL^−1^. The culture medium used was L-15 without phenol red supplemented with 5–10% FBS (v/v) and 1% penicillin/streptomycin (Thermo Fisher, 10,000 IU/mL^−1^ penicillin, 10,000 μg/mL^−1^ streptomycin) at 19 °C. Cell viability was tested by the MTT (tetrazolium salt (2-(4,5-dimethyl-2-thiazolyl)-3,5-diphe-nyl-2H-tetrazolium bromide) method to ensure that the cell cultures are in good condition for carrying out bioaccumulation assays. Toxicity tests were performed with an initial cell amount of 9.0 × 10^5^ cells (volume of 3.0 mL and 3.0 × 10^5^ cell mL^−1^).

Cell toxicity assays (24-h exposure) were performed under sterile conditions in a type II laminar flow cabinet by adding to each well 1.0 mL of cell culture and 3.0 mL of NP dispersions (experiment in duplicate) at several concentrations (dilutions with the cell culture medium). First NP dispersions were redispersed by sonication (10 min, 50% amplitude, pulse 15-s on/10-s off) for citrate-TiO_2_ NPs, and by vortexing for PVP-Ag NPs. After exposure, the cells were transferred to 15-mL tubes for further centrifugation (1500 g, 10 min), and the supernatant was removed. The pellet was mixed with 1.0 mL of freezing medium (Leibovitz medium (L15) with 20% fetal bovine serum (FBS) and 10% DMSO) to preserve the integrity of the cells during freezing and was frozen at −20 °C until analysis.

The obtained cells were further subjected to two different pre-treatments for leading scICP-MS and TEM analysis. Cells for scICP-MS analysis were suspended with 4.0 mL of L15/20% FBS/10% DMSO freezing mixture and were then frozen and kept at −20 °C. Pre-treatment for TEM analysis consisted of a centrifugation stage of the cell suspension at 320 g, 4 °C for 5.0 min and careful supernatant removal, followed by dropwise addition of 500 μL of Karnovsky fixative and keeping the mixture at 4 °C 24 h under gentle shaking. After Karnovsky fixative removal, the pellet was washed with sodium cacodylate buffer (0.1 M) for 20 min (two washing steps) and was kept at 4 °C.

#### Cell suspension preparation for scICP-MS

Cell suspensions were thawed and homogenised through pipette mixing, and then an aliquot was sampled and re-suspended in 1.0% (wt/v) PBS in 1.0-mL Eppendorf tubes. The diluted cell suspensions were subjected to a short and gentle centrifugation washing step (300 g, 4.0°C, 5.0 min). The supernatant was removed, and the pellet was again re-suspended in 1.0% (wt/v) PBS for scICP-MS analysis after appropriate dilution.

#### Microwave-assisted acid digestion

For comparison purposes, 1.0 mL of cell suspensions in 1.0% (wt/v) PBS (one replicate for each case) was subjected to microwave-assisted acid digestion by using 2.0 mL of ultrapure water, 3.0 mL of 69% nitric acid, and 1.0 mL of 33% hydrogen peroxide. The mixtures were exposed to microwave under a controlled temperature program that consisted of a ramp from room temperature to 130 °C in 20 min, and a hold stage at 130 °C for 15 min. After cool down, the acid digests were diluted to 25 mL with ultrapure water and kept at room temperature until ICP-MS analysis (operating conditions given in Table S1, electronic supplementary information, ESI).

#### TEM and SEM analyses

The fixated pellets of clam cells were processed following a routine methodology for TEM. Briefly, the cell pellets were post-fixated in a 1% osmium tetroxide solution. Then, they were dehydrated with increasing ethanol from 50 to 100% and with a final emersion step in propylene oxide. The infiltration was performed using mixtures of propylene oxide: epoxy resin (EMBed-812 kit) at different proportion increasing the amount of resin until having finally pure epoxy resin. Fragments of each pellet embedded were placed in the edge of a silicon template forming blocks. The blocks were cured at 60 °C for 3 days. Ultrathin sections (≈70 nm thick) were prepared using an ultramicrotome with a diamond knife (Diatome) and placed on formvar/carbon-coated 200-mesh copper grids for cells exposed to citrate-TiO_2_ NPs and carbon-coated 400-mesh titanium grids for cells exposed to PVP-Ag NPs to be analysed by TEM.

The fixated pellets of sea bream cells were washed in cacodylate buffer and subsequently filtered through 2-μm polycarbonate membrane. Cell-supported polycarbonate filters were placed on pin stubs (standard 12.7 mm, 8-mm pin length, Ted Pella) and subsequently, they were coated with conductive carbon for SEM analysis.

#### Single-cell ICP-MS measurements

Daily performance for ICP-MS was assessed (Be intensity > 2500 counts s^−1^, In intensity > 40,000 counts s^−1^, U intensity > 30,000 counts s^−1^, and Bkgd ≤ 3 for standards at 1.0 μg L^−1^, and Ce^++^/Ce ratio ≤ 0.05 and the CeO/Ce ratio ≤ 0.025). Daily performance (torch alignment and voltages) was performed with Ti (10 μg L^−1^) and Ag (3.0 μg L^−1^) prior to analysis. Analytical results were calculated using Syngistix™ ICP-MS 2.5 version software. Operating conditions for scICP-MS are listed in Table 1. The scICP-MS analytical data was processed using an iterative approach previously described [21, 35] aiming the cell event threshold. The transport efficiency (TE%) was automatically calculated after measuring the 49.6-nm Au NPs certified reference material at 1.00 × 10^5^ NPs mL^−1^ by the particle frequency method. The TE% value obtained was from 45 to 55%.

**Table 1:** Operating conditions and data acquisition parameters for scICP-MS*

RF power (W)	1600
Plasma gas flow rate (L min^−1^)	15
Make up gas flow rate (L min^−1^)	0.70
Nebulizer gas flow rate (L min^−1^)	0.35
Sample flow rate (μL min^−1^)	10
Quadrupole ion deflector (V)	Set for maximum ion transmission
Triple cone equipment	Sampler, skimmer, and hyperskimmer cones made of nickel
Transport efficiency (%)	≈ 45–55%
Scan time (s)	100
Sample loop (μL)	100
Analyte (m/z)	Ag (107)
Density (g cm^−3^)	10.49
Mass fraction	100%
Mode	Standard
Dwell time (μs)	50
Analyte (m/z)	Ti (131)
Density (g cm^−3^)	4.23
Mass fraction	59.90%
Dwell time (μs)	100
Mode	Reaction cell
Ammonia flow rate (mL min^−1^)	0.75
Ion-product registered	^48^Ti(NH)(NH_3_)_4_
Rejection parameter *q*	0.20

*Quadrupole ion deflector and x/y torch position were set for maximum ion transmission (Ti or Ag)

Determinations implied external calibrations at five level concentrations for ionic titanium (0.5–10 μg L^−1^) for titanium assessment, whereas calibrations with Ag NPs (20, 40, and 60 nm, 1.0 μg L^−1^, each one) were used for silver determinations (Ag NP dispersions were manually shaken just before measurements). Cell suspensions (after dilution in PBS) were placed in 1.0-mL cuvettes of the autosampler. The autosampler automatically mixes the cell suspensions to favour the representativeness of the sample taken and aspirates 150 μL for filling a 100-μL loop. The loaded sample is then pumped at 10 μL min^−1^ and nebulized with a CytoNeb nebulizer coupled to an Asperon chamber which allows a tangential flow that prevents cell damage, collisions, or cell deposition onto the chamber walls. After each injection, the sample loop is rinsed with a wash solution (1% nitric acid and 2% hydrogen peroxide).

Standard mode and mass-shift approach were used for the measurement of silver and titanium, respectively. Ammonia clusters of mass-charge ratio 131 (1.0 mL min^−1^ of ammonia and RPq 0.2 value [33, 34]) were used for titanium determinations. The scICP-MS analytical data were processed using an iterative approach aiming the cell event threshold. Data, graphs, and spectra analysis were performed after data being exported from 2.5 Syngistix ICP-MS software (Single Cell Application). The amount of titanium and silver in cells was determined by applying Eq. (1).

1 $${m}_c=\frac{\varepsilon \cdot {Q}_{\textrm{sam}}\cdot {t}_{\textrm{dwell}}\cdot \left({I}_c-{I}_{bgd}\right)}{m}$$

where *m*_*c*_ is the mass of element of interest in single cell, *ε* is the transport efficiency, *Q*_sam_ is the sample uptake rate, *t*_dwell_ is the dwell time, *m* is the calibration slope, and *I*_*c*_ and *I*_*bgd*_ are the analyte and background intensity, respectively.

## Discussion and results

### Optimization study

The influence of several parameters affecting scICP-MS, such as dwell time and cell concentration, has been evaluated for assessing the content of TiO_2_ NPs and Ag NPs in PVP-100nm Ag NPs–exposed and citrate-45nm TiO_2_ NPs–exposed cells culture from clam gill, sea bass kidney, and sea bream kidney. Other parameters such as those involved in reaction cell (use of ammonia as a reaction gas) when measuring titanium have also been carefully optimised. To date, DRC technology has scarcely been used for single-cell analysis (one development based on oxygen as a reaction gas [29]) and special consideration must be taken since the minimal sample flow rates (10 μL min^−1^) used in scICP-MS. In addition, the obtained signals by scICP-MS could be attributed to cells that contain NPs, cells in which the NPs are attached to membranes, and NPs in the extracellular matrix. Therefore, a washing process is required for removing NPs not tightly attached to cells, and the effect of a washing process (number of washing cycles) has been also evaluated.

Finally, we must consider the existence of NP disaggregation (citrate-TiO_2_ NPs and PVP-Ag NPs conversion into smaller NP aggregates or single NP) and dissolution (citrate-TiO_2_ NPs and PVP-Ag NPs conversion into ionic species) processes, and signals derived from cells could not exclusively been attributed to the citrate-TiO_2_ NPs and PVP-Ag NPs used in the exposure experiments.

#### Dwell time

The dissolved metal content (background signal and extracellular content) and the mean intensity related to the mean mass per cell have been recorded at several dwell times (20, 50, 100, and 200 μs for silver, and 50, 100, and 200 μs for titanium) for cell suspensions from sea bass kidney (1:3000 dilution from cell suspensions at 9.0 × 10^5^ cells mL^−1^) previously exposed to PVP-Ag NPs (100 nm, 100 mg L^−1^) and cell suspensions from clam gills (1:2000 and 1:200 dilution from cell suspensions at 9.0 × 10^5^ cells mL^−1^) previously exposed to citrate-TiO_2_ NPs (45 nm, 100 mg L^−1^). Regarding PVP-Ag NPs (Figure S1,ESI), the average intensity due to the mass of internalised PVP-Ag NPs remains constant at both low and high dwell time values. Small dwell times are preferable to define the peak of cellular events in scICP-MS since low background signals are recorded and the occurrence of double cell peaks (maximising the number of detected events) is minimised [27, 36]. Therefore, good results have been obtained for dwell time of 20 and 50 μs (Figure S2, ESI), and a dwell time of 50 μs has been chosen for further silver determinations.

Regarding titanium assessment (1:1200 and 1:200 dilutions), mean intensity due to the mass of internalised citrate-TiO_2_ NPs (Figure S3, ESI) was found to be similar for dwell times of 50 and 100 μs, but it was slightly higher when recording at 200 μs. These findings can be explained considering the use of reaction cell technology for interference removal for the determination of titanium. The use of ammonia has been reported to broaden the transient signals in spICP-MS [37] leading to tailoring peaks, and a proper recording of the signals is achieved by using high dwell times (100 μs or even 200 μs). However, large dwell times could lead to double event counting, and a dwell time of 100 μs was finally selected for titanium determination (value in accordance with spICP-MS developments when using ammonia as a reaction gas in the reaction cell [34]).

#### Number of washing cycles

**Fig. 1:**
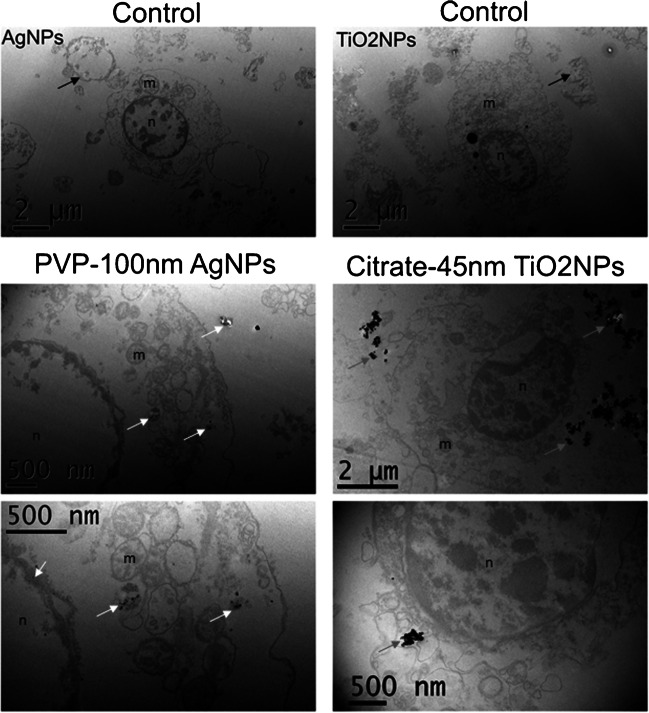
Representative TEM images of non-exposed (controls), PVP-100nm Ag NPs–exposed, and citrate-45nm TiO_2_ NPs–exposed cell cultures from clam gills. The cell cultures were exposed to 50 μg mL^−1^ of PVP-100nm Ag NPs and 5.0 μg mL^−1^ of citrate-45nm TiO_2_ NPs. Black arrows indicate cell debris; grey arrows indicate TiO_2_ NP agglomerates associated to membranes out of the cells; white arrows indicate possible Ag NPs out and inside the cells (of lowers size than the primary NPs). m, mitochondria; n, nucleus

**Fig. 2:**
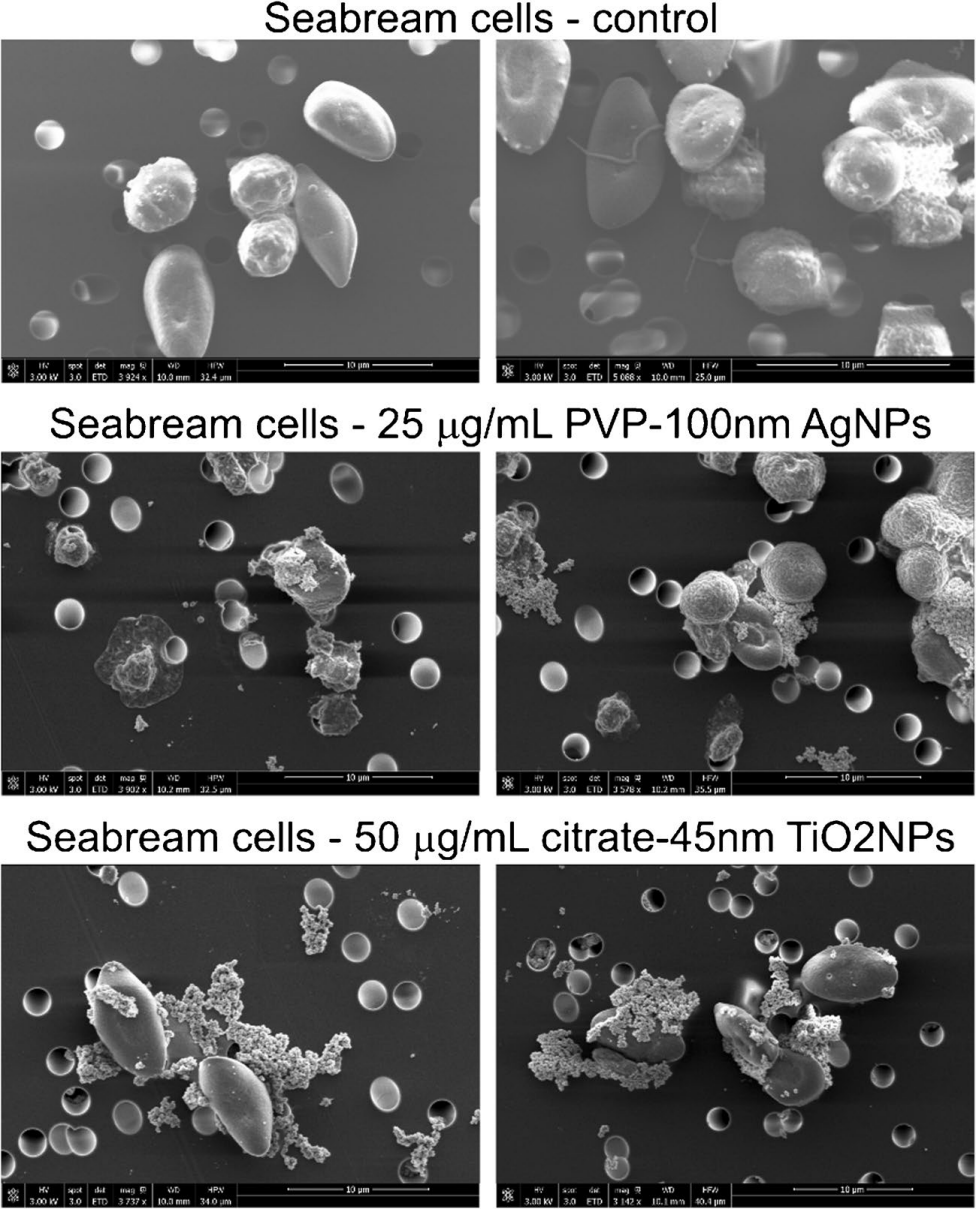
Representative SEM images for sea bream cells exposed to no NPs (control); 100-nm Ag NPs, 25.0 μg mL^−1^; and 45-nm TiO_2_ NPs, 50.0 μg mL^−1^

After cell line exposure to NPs (“*Cell culture conditions and pre-treatments for scICP-MS and EM analysis*” section) non-associated NPs and NPs derived from cell lysis can be present in the extracellular medium of the cell suspensions, and the direct analysis could lead to count non-associated NPs together with cells. The characterization of the NPs-cell interaction by TEM and SEM shows that the NPs not only internalized, but also associated strongly to the cellular membrane. The morphology and ultrastructure of the non-exposed cells observed by TEM indicated that hemocytes [38] are the predominant cells (Figure 1, controls). In addition, TEM images show that citrate-TiO_2_ NPs are mostly adsorbed on the cellular membrane, while cellular uptake of PVP-Ag NPs is observed (Figure 1). Moreover, SEM images of the non-exposed cell samples show features with similar or smaller size and different shape to the cells, which could be attributed to cell debris or apoptotic vesicles (Figure 2, controls), while SEM images of the exposed cell samples show big NP aggregates, which seem to be closely associated to the cell membranes (Figure 2). It is worth to mention that great part of the cells observed in the SEM were blood cells such as blood erythrocytes and lymphocytes [39], which are extensively present in the kidney [40].

The overestimation due to the presence of non-tightly associated NPs to cells can be overcome, in part, by performing a washing procedure with an appropriate medium under gentle centrifugation (cell pellet separation from the bulk solution). Gentle centrifugation conditions must be applied to avoid cell damage, but the procedure must guarantee a minimum amount of cells remaining in the supernatant. Longer centrifugation times would result in a more compact pellet, but it would lead to a more difficult re-suspension of the cell pellet. Therefore, short centrifugation times (within the 3.0–15 min range) at relative centrifugal field (RCF) of 150 and 300 have been commonly used [41–43].

The number of washing cycles (one, two, and three) using 1.0 mL of 1.0% (wt/v) PBS pH 7.4 as a washing buffer and centrifugation at 300 RFC and 4 °C for 5.0 min was studied for cell suspensions from sea bass kidney (1:12,000 and 1:3000 dilution from cell suspensions at 9.0 × 10^5^ cells mL^−1^) previously exposed to PVP-Ag NPs (100 nm, 100 mg L^−1^) and cell suspensions from clam gills (1:1200 and 1:300 dilution from cell suspensions at 9.0 × 10^5^ cells mL^−1^) previously exposed to citrate-TiO_2_ NPs (45 nm, 100 mg L^−1^). The supernatant obtained and the cell pellets after re-suspension in 1.0% (wt/v) PBS were analysed by scICP-MS.

**Fig. 3:**
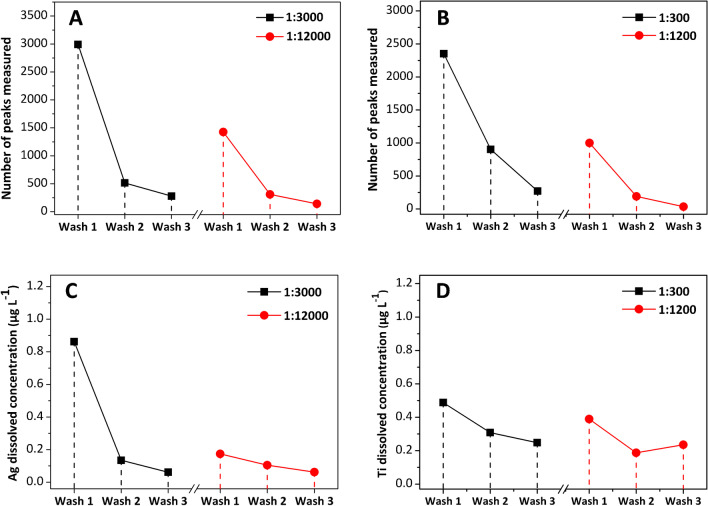
Number of measured peaks for silver and dissolved silver concentration (**A** and **C**, respectively), and number of measured peaks for titanium and dissolved titanium concentration (**B** and **D**, respectively) in the washing buffer (PBS) after several washing cycles. Experiments performed with cell suspensions from sea bass kidney previously exposed to PVP-Ag NPs (100 nm, 100 mg L^−1^) and cell suspensions from clam gills previously exposed to citrate-TiO_2_ NPs (45 nm, 100 mg L^−1^)

Results regarding supernatant (washing fractions) analysis (Figure 3) show a high number of signals (around 3000 in Figure 3A and 2500 in Figure 3B for silver and titanium, respectively) and low ionic silver and titanium concentrations (lower than 1.0 μg L^−1^, Figure 3C, D after performing the first washing cycle, whereas the number of signals decreases considerably after the second and the third washing cycles and the ionic silver and titanium concentration remains constant. These findings suggest that most of the free NPs are efficiently removed during the first washing stage, and one washing cycle with 1.0 mL of 1.0% (wt/v) PBS and centrifugation (300 RFC, 4 °C) for 5.0 min was chosen.

Finally, Figure S4 (ESI) shows TEM images of clam gill cells exposed to PVP-15nm Ag NPs (5 mg L^−1^) and to citrate-45nm TiO_2_ NPs (5 mg L^−1^). PVP-Ag NPs were found to be removed from the extracellular medium (they were found to be attached to the inner membrane surface or just phagocytised), but citrate-TiO_2_ NPs were found to remain in part as quite large agglomerates in the extracellular medium and may not have been totally removed during the washing steps of the centrifugation (similar or higher weight than the cells, implying their settlement in the cell pellet).

#### Cellular concentration

Data from the available scICP-MS literature has shown that the cell concentration should not change the average mass per cell measured, and only the frequency (number) of signals will be dependent on the cell concentration [18, 36]. High dilution could therefore be needed for trials carried out at high NP concentrations and/or long exposure times for avoiding saturation in the detector because of the large number of incorporated nanoparticles (high mass of the element per cell).

Different dilutions of cell suspensions from sea bass kidney or clam gills (9.0 × 10^5^ cells mL^−1^) after exposure previously exposed to 100-nm PVP-Ag NPs (nominal mass concentration at 100 mg L^−1^) and 45-nm citrate-TiO_2_ NPs (nominal mass concentration at 100 mg L^−1^) have been tested (experiments in triplicate), and Figure S5 (ESI) shows that the mean mass is within the 10–12 fg per cell range for silver (1:3000 to 1:30,000 dilution), and from 40 to 45 fg per cell for titanium (1:300 to 1:9000 dilution). The small differences observed can be attributed to the scICP-MS analysis variability itself. Results are quite convenient since the cell concentration will be strongly influenced by the cell and NPs’ nature, and the exposure conditions (NP concentration and exposure time) and dilutions can be performed without information losses.

### Comparison of scICP-MS with microwave-assisted acid digestion and ICP-MS

The amount of cell-associated citrate-TiO_2_ NPs and PVP-Ag NPs in selected cells isolated from tissues after NP exposure was assessed by the proposed scICP-MS method and after applying a conventional microwave-assisted acid digestion process and direct ICP-MS analysis. Selected cell samples consisted of cells isolated from clam gills exposed to 5-nm citrate-TiO_2_ NPs at 50 μg mL^−1^ and to 25-nm citrate-TiO_2_ NPs at 50 and 100 μg mL^−1^; cells isolated from sea bass and sea bream kidneys exposed to 25-nm citrate-TiO_2_ NPs at 50 μg mL^−1^; cells isolated from clam gills exposed to 15-nm PVP-Ag NPs at 100 μg mL^−1^; cells isolated from sea bass kidney exposed to 100-nm PVP-Ag NPs at 100 μg mL^−1^; and cells from sea bream’s kidney exposed to 15-nm PVP-Ag NPs at 100 μg mL^−1^. For comparison (concentrations expressed as femtograms per cell), the total titanium and silver content obtained by ICP-MS after microwave-assisted acid digestion was divided by the number of cells counted by cytometry (9.24 × 10^4^ cells). Results are listed in Table 2 and slightly higher concentrations (about 1.3 to 3.6 times higher) were obtained when using ICP-MS after microwave-assisted acid digestion when analysing clam cells for citrate-TiO_2_ NPs. High differences were also obtained for cells isolated from both fish kidneys (about 3.3 to 9.0 times higher) for citrate-TiO_2_ NP assessment. In the case of PVP-Ag NPs, higher differences were observed: 6.4 times higher for clam gill cells and 10 to 17 times higher for sea bass and sea bream cells. Reports that focused on studying the internalization of dissolved metal/metalloid have revealed that concentrations by ICP-MS after acid digestion are slightly higher than those obtained by scICP-MS, about 1.3 times higher for copper in human red blood cells [18], and about 3 times higher for total carbon and platinum in cell samples [41]. Differences, also reported by Meyer et al. [17] for arsenite assessment in A549 cells, have been attributed to a lower number of cells involved in scICP-MS measurements. Regarding experiments for NPs associated with cells, López-Serrano et al. [26] have reported that the lower element concentration when using scICP-MS could be also attributed to saturation of the detector when the cells contain a high number of NPs (around 100 per cell in the case of Ag NPs in THP-1 monocytes). The saturation of the detector may be the most possible explanation for the differences found in our experiments because cells exposure tests have been conducted at high NP concentrations.

**Table 2:** Titanium and silver concentrations in some cell lines exposed to PVP-Ag NPs and citrate-TiO_2_ NPs after microwave-assisted acid digestion and ICP-MS measurement and after spICP-MS

Sample ID	[Ti](femtograms per cell)^a^	RSD	[Ti](femtograms per cell)^b^	RSD	Ratio^c^
Clam (5.0-nm citrate-TiO_2_ NPs, 50 μg mL^−1^)	146	2	41	5	3.6
Clam (25-nm citrate-TiO_2_ NPs, 50 μg mL^−1^)	167	8	47	1	3.5
Sea bass (5.0-nm citrate-TiO_2_ NPs, 50 μg mL^−1^)	873	8	35	-	25
Sea bass (25-nm citrate-TiO_2_ NPs, 50 μg mL^−1^)	152	13	46	4	3.3
Sea bream (5.0-nm citrate-TiO_2_ NPs, 50 μg mL^−1^)	562	2	57	1	9.9
Sample ID	[Ag](femtograms per cell)^a^	RSD	[Ag](femtograms per cell)^b^	RSD	Ratio^c^
Clam (15-nm PVP-Ag NPs, 5.0 μg mL^−1^)	69	4	1.9	5	37
Clam (100-nm PVP-Ag NPs, 5.0 μg mL^−1^)	24	9	38	-	6.4
Sea bass (100-nm PVP-Ag NPs, 100 μg mL^−1^)	367	16	21	7	17
Sea bream (100-nm PVP-Ag NPs, 5.0 μg mL^−1^)	147	10	16	6	9.7

^a^Microwave-assisted acid digestion and ICP-MS measurement; ^b^spICP-MS measurement; ^c^ratio between the concentrations after microwave-assisted acid digestion and ICP-MS measurement and spICP-MS measurement

### Limit of detection of the method

The limits of detection (LODs), referred to the mass of element (titanium and silver) associated with cells, have been automatically obtained with the NanoSyngistix™ ICP-MS software after establishing the transport efficiency and dissolved titanium calibrations within 0.5–10 μg L^−1^, or calibrations based on Ag NPs (nominal diameters of 20, 40, and 60 nm, 1.0 μg L^−1^, each one). Calculated values were 0.095 ± 0.011 fg per cell for titanium and 0.005 ± 0.001 fg per cell for silver.

### Applications

Exposure assays for cells isolated from clam gills were performed with 15- and 100-nm PVP-Ag NPs at two concentration levels (5.0 and 50 μg mL^−1^) and with 5.0- and 25-nm citrate-TiO_2_ NPs at 50 μg mL^−1^. Regarding cells from sea bass kidney, exposure assays were carried out with 15- and 100-nm PVP-Ag NPs at 50 and 100 μg mL^−1^; whereas 5.0- and 25-nm citrate-TiO_2_ NPs were used at 50 μg mL^−1^. Finally, 15- and 100-nm PVP-Ag NPs (10, 50, and 100 μg mL^−1^) and 5.0- and 25-nm citrate-TiO_2_ NPs (10 and 50 μg mL^−1^) were tested for sea bream cells. After scICP-MS analysis (dilution between 100 and 600 for titanium, and within the 600–3000 range for silver), the average mass of the element (silver and titanium) per cell, as well as the element concentration in the supernatant from cell washing steps, and the theoretical NP internalization and/or NP adsorption onto the cellular membranes (range-mass levels) are obtained for each case.

**Fig. 4:**
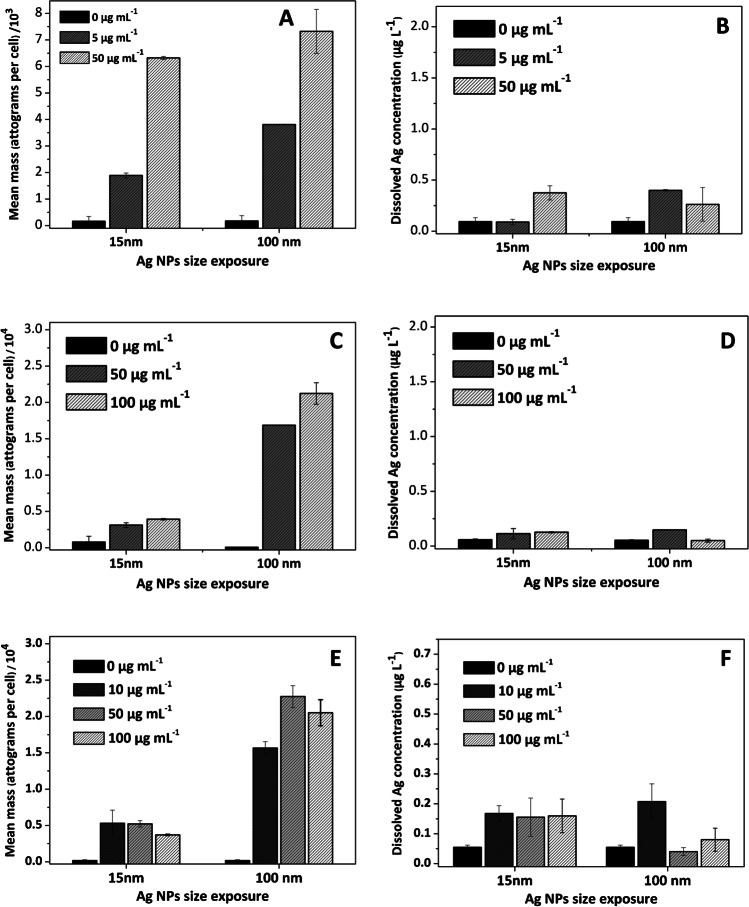
Cellular silver (attograms per cell, **A**, **C**, and **E**) and extracellular (dissolved, **B**, **D**, and **F**) silver concentration in clam gill cells (**A** and **B**), sea bass kidney cells (**C** and **D**), and sea bream kidney cells (**E** and **F**)

**Fig. 5:**
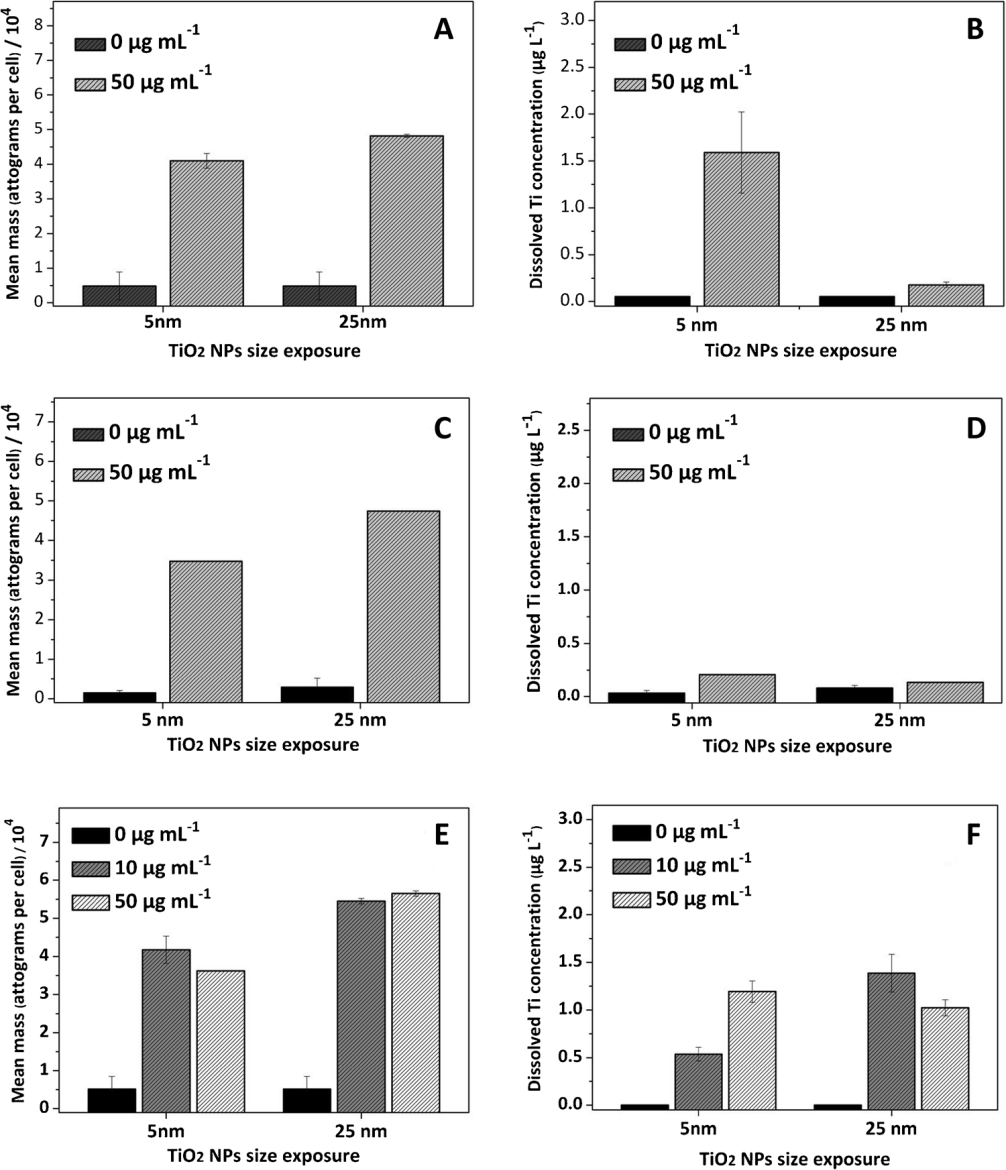
Cellular titanium (attograms per cell, **A**, **C**, and **E**) and extracellular (dissolved, **B**, **D**, and **F**) titanium concentration in clam gill cells (**A** and **B**), sea bass kidney cells (**C** and **D**), and sea bream kidney cells (**E** and **F**)

Results on mean mass per cell and dissolved metal concentration are plotted in Figs 4 and 5 for silver and titanium assessment, respectively. The levels of dissolved silver and titanium in the extracellular medium for the three types of cells were found to be very small (lower than 0.5 μg L^−1^), even for exposure experiments at high PVP-Ag NP and citrate-TiO_2_ NP concentrations.

The mean silver mass per cell in cells from clam gills (Figure 4A) was found to be dependent on the PVP-Ag NP concentration used for the exposure assays, being lower for cells exposed to the lowest PVP-Ag NP concentration independently of the PVP-Ag NP nominal size (15 and 100 nm). Concerning PVP-Ag NPs’ size, there is a big difference between the mean silver mass per cell for experiments using the lowest PVP-Ag NP concentration (5.0 μg mL^−1^, 15 and 100 nm), but the mean silver content per cell is quite similar when the highest concentration (100 μg mL^−1^) was used, and values of 6.3 ± 0.094 and 6.7 ± 0.83 fg per cell were assessed for experiments with 15 and 100 nm, respectively.

Regarding silver interaction with cells isolated from sea bass and sea bream kidneys, a quite different trend has been obtained (Figure 4C), and there is a big difference for silver in the cellular fraction when exposed with 15- and 100-nm PVP-Ag NPs. However, in comparison within the same size, quite similar silver concentrations were obtained, mainly for 15-nm PVP-Ag NPs (3.1 ± 0.32 and 3.9 ± 0.098 fg per cell in sea bass kidney cells for 50 and 100 μg mL^−1^, respectively). In the case of sea bream kidney cells (Figure 4E), quite similar silver concentrations were obtained for 15-nm PVP-Ag NPs (5.3 ± 1.8 and 5.2 ± 0.43 fg per cell for 10 and 50 μg mL^−1^, respectively), but a lower value was assessed when using the highest PVP-Ag NP concentration (3.7 ± 0.092 fg per cell at 100 μg mL^−1^).

Citrate-TiO_2_ NP interaction with cells is also quite different than that observed for PVP-Ag NPs, and quite similar mean titanium mass per cell was obtained independently of the citrate-TiO_2_ NP concentration and size used, and the cell nature (Figure 5A, C, and E for clam gill, sea bream kidney, and sea bass kidney, respectively).

The theoretical number of cellular PVP-Ag NPs is given in Tables S2 to S4 for clam gill cells, sea bass kidney cells, and sea bream kidney cells, respectively. The theoretical approximation considers the measured femtograms for each element (titanium and silver) and values such as density, mass fraction, and radius of the citrate-TiO_2_ NPs and PVP-Ag NPs used for the interaction assays. Therefore, a 100-nm PVP-Ag NP equals to 5.5 fg under this criterion, whereas, for 15-nm PVP-Ag NPs, the calculated femtograms are close to the LOD of the technique for silver (0.005 ± 0.001 fg per cell).

**Fig. 6:**
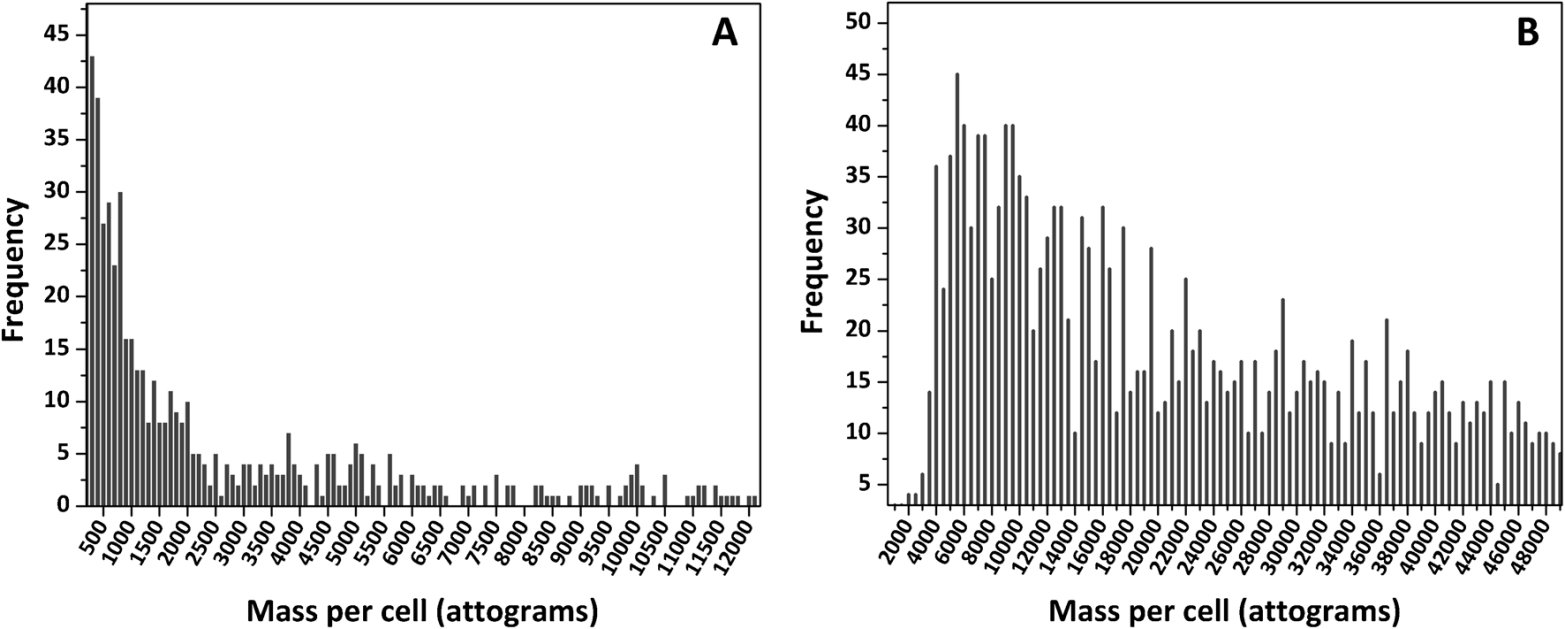
Frequency histograms from sea bream kidney cells after exposure to (**A**) 100-nm PVP-Ag NPs (5.0 μg mL^−1^) and (**B**) 5.0-nm citrate-TiO_2_ NPs (50 μg mL^−1^)

Data, after applying this theoretical calculation and distributing the data obtained in intervals of 5.0 fg up to the upper limit of the technique (5.0 fg), are listed in Tables S2 to S4 (ESI) for cells from clam gills and sea bass and sea bream kidneys exposed to 100-nm PVP-Ag NPs. The number of peaks (number of cells containing silver) appears to be independent on the Ag NP concentration for cells derived from clam gills (1207 and 1187 peaks for 5.0 and 50 μg mL^−1^, respectively) and sea bream kidney (902, 1108, and 959 peaks for 10, 50, and 100 μg mL^−1^, respectively). However, PVP-Ag NP concentration influences the amount of silver in cells from sea bass kidney (1918 and 1256 peaks for 50 and 100 μg mL^−1^, respectively). Table S2 shows that the percentage of PVP-Ag NPs lower than 100 nm is higher in clam gill cells (82 and 47% for 5.0 and 50 μg mL^−1^, respectively) than in sea bream (30, 16, and 19% for 10, 50, and 100 μg mL^−1^, respectively) and sea bass (12 and 7% for 50 and 100 μg mL^−1^, respectively) kidney cells, which could imply a higher dissolution (ionization) of PVP-Ag NPs by clam gill cells. Table S3 (ESI) also shows that the number of (theoretically) internalized 100-nm PVP-Ag NPs is gradually reduced and no more than nine 100-nm Ag NPs per cell can be identified. These results lead to narrow frequency histograms (Figure 6A) where most of the silver refers to few femtograms (dissolved silver, PVP-Ag NPs lower than 100 nm, and a very small amount of 100-nm PVP-Ag NPs).

Regarding titanium, 5.0-nm citrate-TiO_2_ NPs and 25-nm citrate-TiO_2_ NPs lead to theoretical femtograms per cell close/lower than the LOD (0.095 ± 0.011 femtograms per cell). However, titanium in the cellular fraction has been assessed because of the high degree of agglomeration of citrate-TiO_2_ NPs. Therefore, Tables S5 to S7 (clam gill cells, sea bass kidney cells, and sea bream kidney cells, respectively) show the range (5.0 intervals) of femtograms per cell (associated titanium) instead of the theoretical number of associated citrate-TiO_2_ NPs. Results show that interaction with clam gill cells appears to be independent on the size of citrate-TiO_2_ NPs (5.0 and 25 nm). Therefore, the highest percentages of cellular titanium in clam gill cells (percentages higher than 10%) have been found for femtograms per cell within the < 5.0 to 15–20 range for both citrate-TiO_2_ NPs sizes (Table S5). Regarding sea bass and sea bream cells (Table S6 and S7, respectively), differences since the citrate-TiO_2_ NP size were attempted, and higher percentages of associated NPs to the cell were obtained for experiments with 5.0-nm citrate-TiO_2_ NPs (31.7% of < 5.0 fg per cell for sea bass kidney, and percentages of 37.2 and 29.7% of < 5.0 fg per cell for sea bream kidney exposed to 50 and 10 μg mL^−1^, respectively). Table S6 and S7 show, therefore, that the degree of citrate-TiO_2_ NP interaction with cells from sea bass and sea bream kidney is dependent on the citrate-TiO_2_ NP size. In addition, the histograms obtained (such as the one shown in Figure 6B) present large frequencies for all the ranges of masses per cell recorded.

## Conclusions

Parameters such as dwell time, cell concentration, and mainly, the presence of NPs in the extracellular medium have been found to condition the reliable assessment of PVP-Ag NPs and citrate-TiO_2_ NP direct interaction with clam and fish kidney cells by scICP-MS. In addition, free-interference titanium determinations have been possible by using ammonia as a reaction gas in the scICP-MS measurement mode. The developed procedure offers great potential for studying NP interactions with cells, including internalization, and results show that the interaction is mainly dependent on the cell nature and the NP type, and the effects of the NP concentration and size is less important. The degree of internalization of citrate-TiO_2_ NPs was found to be lower than that observed for PVP-Ag NPs due to the high degree of agglomeration of citrate-TiO_2_ NPs (TEM analysis has revealed citrate-TiO_2_ NP interactions with the external part of cell membranes as well as citrate-TiO_2_ NP agglomerates in the extracellular fluid). On the other hand, the scICP-MS results for both the silver and titanium contents show significant differences with those found after acid digestion of the cells and direct ICP-MS measurement, results that agree with those reported in the literature and that in our case may be due to the high nanoparticle concentrations used in the cell exposure trials.

## Supplementary Information

ESM 1 (DOCX 935 kb)
