# Supplementary material for: Single-cell ICP-MS for studying the association of inorganic nanoparticles with cell lines derived from aquaculture species
Source: Anal Bioanal Chem. 2023 May 10;415(17):3399–413. doi: 10.1007/s00216-023-04723-6 (PMC10289938; doi:10.1007/s00216-023-04723-6)
Supplement: Supplementary file 1 — (DOCX 935 kb) [file 216_2023_4723_MOESM1_ESM.docx]

**Electronic supplementary information**

**Single-cell-ICP-MS for studying the association of inorganic nanoparticles with cell lines derived from aquaculture species**

Cristian Suárez-Oubiña, Paloma Herbello-Hermelo, Natalia Mallo, María Vázquez, Santiago Cabaleiro, Ivone Pinheiro, Laura Rodríguez-Lorenzo, Begoña Espiña, Pilar Bermejo-Barrera, Antonio Moreda-Piñeiro

**Figure S1**. Representative TEM images of (A) Citrate-5nm TiO_2_ NPs, (B) Citrate-45nm TiO_2_ NPs, (C) PVP-15nm Ag NPs, and (D) PVP-100nm Ag NPs. High-resolution TEM analysis of citrate-5nm TiO_2_ NPs was performed using FEI Titan (G3) Cubed Themis 60-300 kV electron microscope, operating at 200 kV. TEM images of the rest of NPs were acquired using JEOL JEM 1010 transmission electron microscope operating at 100 kV.

**Table S1**. Operating ICP-MS conditions

| Spray Chamber Type | Quartz Cyclonic |
| --- | --- |
| PC^3x^ Peltier Cooler System | 4ᵒC |
| Nebulizer Type | PFA MicroFlow |
| RF power (W) | 1600 |
| Plasma Gas Flow (L min^-1^) | 15 |
| Auxiliary Gas flow (L min^-1^) | 1.2 |
| Nebulizer Gas Flow (L min^-1^) | 1.14 |
| Sample uptake rate (µL min^-1^) | ≈ 220 |
| Acquisition Mode | Scanning |
| Dwell time per amu (ms) | 50 ms |
| Quadrupole ion deflector (V) | Set for maximum ion transmission |
| Sweeps | 20 |
| Readings | 1 |
| Replicates | 3 |
| Analyte (m/z) | Ag (107)  Standard mode |
| Analyte (m/z) | Ti (131)  DRC mode (0.75 mL min^-1^ ammonia flow rate)  Ion-product registered: ^48^Ti(NH)(NH_3_)_4_  Rejection parameter (q): 0.20 |

**Figure S2**. Effect of the dwell time on the silver mean intensities (n=5). RSD values were lower than 1%.

**Figure S3**. Effect of the dwell time on the titanium mean intensities (n=5). RSD values were lower than 1%.

**Figure S4**. Complementary studies using Transmission Electron Microscopy: (left) cell clams, PVP-15 nm Ag NPs, 5 mg L^-1^ and (right) cell clams, CT-45 nm TiO_2_ NPs, 5 mg L^-1^.

**Figure S5**. Effect of the cells concentration (n=3) on internalised silver (A) and titanium (B) contents. RSD values were lower than 3%.

**Table S2**. Theoretical Ag NPs internalization in mantle clam (*Ruditapes philippinarum*) cells

|  | **100nm, 5.0 µg mL^-1^** | | **100nm, 50 µg mL^-1^** | |
| --- | --- | --- | --- | --- |
| Ag NPs per cell | Found peaks^a^ | Percentage (%) | Found peaks^b^ | Percentage (%) |
| **< 100 nm** | 972 | 81.9 | 555 | 46.8 |
| **1** | 64 | 5.4 | 116 | 9.8 |
| **2** | 25 | 2.1 | 65 | 5.5 |
| **3** | 18 | 1.5 | 57 | 4.8 |
| **4** | 20 | 1.7 | 66 | 5.6 |
| **5** | 17 | 1.4 | 71 | 6.0 |
| **6** | 29 | 2.4 | 60 | 5.0 |
| **7** | 20 | 1.7 | 57 | 4.8 |
| **8** | 30 | 2.5 | 81 | 6.8 |
| **9** | 12 | 1.0 | 59 | 5.0 |
| (a) Total number of peaks = **1207**: (b) Total number of peaks = **1187** | | | | |

**Table S3**. Theoretical Ag NPs internalization in sea bass (*Dicentrarchus labrax*) kidney cells

|  | **100nm, 50 µg mL^-1^** | | **100nm, 100 µg mL^-1^** | |
| --- | --- | --- | --- | --- |
| NPs per cell | Found peaks^a^ | Percentage (%) | Found peaks^b^ | Percentage (%) |
| **<100nm** | 223 | 11.6 | 85 | 6.8 |
| **1** | 176 | 9.2 | 130 | 10.3 |
| **2** | 238 | 12.4 | 184 | 14.6 |
| **3** | 220 | 11.5 | 156 | 12.4 |
| **4** | 208 | 10.8 | 153 | 12.2 |
| **5** | 194 | 10.1 | 150 | 11.9 |
| **6** | 184 | 9.6 | 131 | 10.4 |
| **7** | 205 | 10.7 | 109 | 8.7 |
| **8** | 186 | 9.7 | 108 | 8.6 |
| **9** | 84 | 4.4 | 50 | 4.0 |
| (a) Total number of peaks = **1918**: (b) Total number of peaks = **1256** | | | | |

**Table S4**. Theoretical Ag NPs internalization in sea bream bream (*Sparus aurata*) kidney cells.

|  | **100nm, 100 µg mL^-1^** | | **100nm, 50 µg mL^-1^** | | **100nm, 10 µg mL^-1^** | |
| --- | --- | --- | --- | --- | --- | --- |
| NPs per cell | Found peaks^a^ | Percentage (%) | Found peaks^b^ | Percentage (%) | Found peaks^c^ | Percentage (%) |
| **<100nm** | 178 | 18.6 | 159 | 15.8 | 271 | 30.0 |
| **1** | 111 | 11.6 | 110 | 10.9 | 102 | 11.3 |
| **2** | 84 | 8.8 | 99 | 9.8 | 83 | 9.2 |
| **3** | 98 | 10.2 | 83 | 8.2 | 86 | 9.5 |
| **4** | 71 | 7.4 | 77 | 7.6 | 86 | 9.5 |
| **5** | 84 | 8.8 | 117 | 11.6 | 65 | 7.2 |
| **6** | 67 | 7.0 | 86 | 8.5 | 49 | 5.4 |
| **7** | 84 | 8.8 | 85 | 8.4 | 52 | 5.8 |
| **8** | 95 | 9.9 | 101 | 10.0 | 64 | 7.1 |
| **9** | 87 | 9.1 | 91 | 9.0 | 44 | 4.9 |
| (a) Total number of peaks = **959**: (b) Total number of peaks = **1108**; (c) Total number of peaks = **902** | | | | | | |

**Table S5**. Theoretical TiO_2_ NPs internalization in mantle clam (*Ruditapes philippinarum*) cells.

|  | **25nm, 50 µg L^-1^** | | **5nm, 50 µg L^-1^** | |
| --- | --- | --- | --- | --- |
| Femtograms  per cell | Found peaks^a^ | Percentage (%) | Found peaks^b^ | Percentage (%) |
| 1.0 – 5.0 | 102 | 14.6 | 551 | 18.0 |
| 5.0 - 10 | 82 | 11.7 | 585 | 19.1 |
| 10 - 15 | 87 | 12.4 | 399 | 13.0 |
| 15 – 20 | 77 | 11.0 | 347 | 11.3 |
| 20 - 25 | 67 | 9.6 | 280 | 9.1 |
| 25 – 30 | 71 | 10.1 | 227 | 7.4 |
| 30 - 35 | 57 | 8.1 | 193 | 6.3 |
| 35 – 40 | 62 | 8.9 | 185 | 6.0 |
| 40 - 45 | 58 | 8.3 | 164 | 5.3 |
| 45 - 50 | 37 | 5.3 | 138 | 4.5 |
| (a) Total number of peaks = **934**: (b) Total number of peaks = **3132** | | | | |

**Table S6**. Theoretical TiO_2_ NPs internalization in sea bass (*Dicentrarchus labrax*) kidney cells.

|  | **25nm, 50 µg L^-1^** | | **5nm, 50 µg L^-1^** | |
| --- | --- | --- | --- | --- |
| Femtograms  per cell | Found peaks^a^ | Percentage (%) | Found peaks^b^ | Percentage (%) |
| 1.0 – 5.0 | 136 | 14.6 | 1056 | 31.7 |
| 5.0 - 10 | 111 | 11.9 | 533 | 16.0 |
| 10 - 15 | 111 | 11.9 | 381 | 11.4 |
| 15 – 20 | 91 | 9.7 | 333 | 10.0 |
| 20 - 25 | 85 | 9.1 | 259 | 7.8 |
| 25 – 30 | 93 | 10.0 | 201 | 6.0 |
| 30 - 35 | 82 | 8.8 | 191 | 5.7 |
| 35 – 40 | 89 | 9.5 | 157 | 4.7 |
| 40 - 45 | 77 | 8.2 | 118 | 3.5 |
| 45 - 50 | 59 | 6.3 | 103 | 3.1 |
| (a) Total number of peaks = **934**: (b) Total number of peaks = **3332** | | | | |

**Table S7**. Theoretical TiO_2_ NPs internalization in sea bream (*Sparus aurata*) kidney cells.

|  | **25nm, 10 µg L^-1^** | | **25nm, 50 µg L^-1^** | | **5nm, 10 µg L^-1^** | | **5nm, 50 µg L^-1^** | |
| --- | --- | --- | --- | --- | --- | --- | --- | --- |
| Femtograms  per cell | Found peaks^a^ | Percentage (%) | Found peaks^b^ | Percentage (%) | Found peaks^c^ | Percentage (%) | Found peaks^d^ | Percentage (%) |
| 1.0 – 5.0 | 41 | 13.4 | 50 | 6.3 | 903 | 29.7 | 817 | 37.2 |
| 5.0 - 10 | 28 | 9.1 | 67 | 8.4 | 561 | 18.4 | 345 | 15.7 |
| 10 - 15 | 30 | 9.8 | 70 | 8.8 | 391 | 12.9 | 234 | 10.7 |
| 15 – 20 | 30 | 9.8 | 71 | 8.9 | 277 | 9.1 | 151 | 6.9 |
| 20 - 25 | 31 | 10.1 | 94 | 11.8 | 242 | 8.0 | 149 | 6.8 |
| 25 – 30 | 32 | 10.4 | 84 | 10.5 | 175 | 5.8 | 129 | 5.9 |
| 30 - 35 | 31 | 10.1 | 92 | 11.5 | 169 | 5.6 | 106 | 4.8 |
| 35 – 40 | 33 | 10.8 | 99 | 12.4 | 133 | 4.4 | 110 | 5.0 |
| 40 - 45 | 24 | 7.8 | 88 | 11.0 | 125 | 4.1 | 80 | 3.6 |
| 45 - 50 | 27 | 8.8 | 82 | 10.3 | 67 | 2.2 | 75 | 3.4 |
| (a) Total number of peaks = **307**; (b) Total number of peaks = **797**: (c) Total number of peaks = **3043**; (d) Total number of peaks = **2196** | | | | | | | | |
